# "The Hospital" Nursing Mirror

**Published:** 1900-07-28

**Authors:** 


"The Hospital" Nursing Mirror.

Being the Nursing Section of "The Hospital."

[Contributions for this Section of "The Hospital" should be addressed to the Editor, The Hospital, 28 & 29, Southampton Street, Strand, London, W.C., and should have the word "Nursing" plainly written in left-hand top corner of the envelope.]

### Notes on News from the Nursing World.

### THE RED CROSS SOCIETY.

A Sister of the A.N.S.R. at a Bloemfontein hospital writes: "You will, I am sure, be glad to hear what a boon we find the Red Cross Society. You can get any mortal thing you want within reason from them, and poor, sick Tommy, when he leaves the hospital, goes out, owing to their care, well clad in warm clothes, with a pipe of tobacco into the bargain. Only to-day I got six pipes and some cakes of tobacco for my men. The very bad patients are able to have champagne, without which they would undoubtedly die."

### NURSES AND CHINA.

A number of our readers inquire whether female nurses are wanted for service in China, and some of them ask for particulars respecting the climate of the country. It depends upon circumstances how many nurses will be needed, but we assume that the Army Medical Department do not propose, like the authorities of the "Maine," to confine themselves to male nurses. Application should be made to the Department, 18, Victoria Street, S.W., but admission to the Army Nursing Reserve is an indispensable preliminary to service under the auspices of the War Office. The climate of China varies greatly. In some parts the heat in summer is intense, and in others the cold in winter is bitter. But it is highly improbable that nurses will be sent to the interior.

### MR. WATSON CHEYNE ON NURSING AT THE FRONT.

In the course of a long letter describing his experiences as consulting surgeon to the army in South Africa, Mr. Watson Cheyne alludes to the question of nursing. He "strongly agrees" that female nurses are better, "especially for cases of typhoid fever" than males. Admitting that women could not go forward with an advancing army, and deprecating their employment in field hospitals, he holds that female nurses must be limited to base hospitals. Mr. Watson Cheyne proceeds:—

"As a matter of fact, in this campaign a much larger number of female nurses than is arranged for in the Army Regulations was employed. My belief is that a still larger number might have been used with advantage, but even if the authorities had taken the same view any attempt to substitute female nurses for the orderlies to such an extent as is the case in a civil hospital would have dislocated arrangements most seriously, and would have meant practically the reorganisation of the base hospital system in the middle of the campaign when the officials were already very much overworked. This is, however, a point which I think should be kept in view when the numerous lessons taught by the war are taken into consideration."

### NURSING IN NATAL.

The following reaches us from a nurse at one of the stationary hospitals in Natal, under date June 26th: "We have had another move since last I wrote. We came up here as soon as ever we could get along the railway. It is no small job to move a hospital of 500 beds. We occupied in all about four trains with our equipment, cattle, luggage, &c. It was an interesting though a tiring experience. We went through the Biggarsberg and various passes, but it made one very sad to find everything in ruins as we came along. The bridges are all destroyed, so we crossed the rivers on wooden trestles with lines over, and most of the way we could only go at a speed of four miles an hour; it therefore took us from eight a.m. to six p.m. to complete our journey. All the shops and houses are frightfully injured—windows smashed, doors taken off their hinges, and all the wanton damage possible done by the Boers. We have set up a very large camp here, which has involved a lot of hard work. These are all new E.P. tents—they are also called Egyptian or Indian tents—holding eight patients in each, and they will remain if we move on again, as we may do, should the staff of another general hospital come to take our place. In that case, we shall take with us only the small bell-tents. The other night we had a great excitement. The Boers are about in small parties, and were expected down here, so the search-light was round about us all night. The enemy did attack the line, but we were quite safe, as we are surrounded by large camps of troops, amongst whom are nearly 1,000 Highlanders. One afternoon we were invited to tea at their mess, and six of us went. It was such a pleasant change; they sent the ambulance waggon ten miles and three drivers for us. Enteric is as bad as ever; it never seems to get any less, and jaundice is also very prevalent. I suppose it is on account of the chills people get. When the sun is out it is very hot, but at night the cold is intense. The water in our tents is frozen in the morning, and I can never use my sponge, it is so stiff. How we shall enjoy home comforts after this!"

### "SPOILING TOMMY."

The Acting Superintendent, Army Nursing Service, of the hospital ship "Princess of Wales," in an interview which appears in another part of the paper, utters a timely word of warning. She is afraid that "Tommy" is being spoilt too much, and that the discipline of the army will be ruined if care is not taken to show discretion in the attentions which are showered upon him. Not that Miss Chadwick is in the least degree lacking in sympathy. On the contrary, she says that it made her heart ache to see the men when they came on board, "like skeletons, the skin just drawn over the bones." They should, she thinks, have every consideration, "but," she adds, "it is just as bad to overdo it as it is to underdo it." Unfortunately, the happy mean is not always easy of attainment.

### NURSING AT WYNBERG CAMP.

A member of the Army Nursing Service Reserve who has just arrived at the General Hospital, Wynberg, writes us under date of July 1st:—

"We had a most pleasant and enjoyable voyage out on the R.M.S. 'Briton.' There were sixty nursing sisters on board, twenty-five bound for Cape Town, twenty-five for Natal, and ten for the Imperial Yeomanry Hospital. I was amongst the number bound for Cape Town. On arrival there we were sent out to Wynberg, where the sisters welcomed us gladly, as they had been having such a busy time. The hours for sisters on duty here are practically the same as military stations at home, but, of course, when very busy sisters cannot get their off duty time regularly. They all seem to like being here very much, though most would like to be sent nearer to the front. We get large convoys of patients from the front nearly every day, and the preparations made to receive them seem very efficient. As soon as possible all who are fit to travel are sent home to England, and the sick ones get every attention and comfort they could possibly desire. Most of the patients are suffering from enteric, the dysentery does not seem so prevalent at present, and the surgical cases have most of their wounds healed up before they arrive here. We had a lot of men down from Pretoria yesterday; they had been imprisoned there and got enteric. Three invalid sisters have been sent here from Bloemfontein; they will go home on sick leave as soon as they are fit to travel, and we are expecting more down every day now. Eighteen sisters were sent from here to Bloemfontein this week to take the places of sick ones. I am on night duty, and have a very cosy little room and a nice bed with mosquito curtains. I have had several bites, but the mosquitos and flies are not so bad now, as it is winter."

### A SYDNEY NURSE AT BLOEMFONTEIN.

A member of the New South Wales Army Nursing Reserve, writing from Bloemfontein, says: "We are one and all on the coarsest fare and the barest necessaries of life. We cannot get any washing done at all, for we have no soap in the town in any quantity; there is no kerosene, and when we can buy it it is 19s. a tin; butter, when you can buy it, is 5s. 6d. lb.; but we don't get it. We just roll up in our blankets and get our rest anyhow." But she is none the less glad that she is in South Africa, and she declares that the wounded soldiers are patient and uncomplaining. In acknowledging the receipt of articles sent from the New South Wales people for the patients, she mentions that day shirts are much wanted, also plenty of socks, full size; Crimean caps, ink, writing paper, envelopes, razors, and tins of jam. Mosquito nets are very much appreciated.

### A BELGIAN NURSE ON THE BOER SOLDIERS.

Madame Alice Bron, who went out to South Africa as a member of the Belgian Ambulance, which the people of that country despatched to care for the Boer wounded, came away with a very poor opinion of the men to whom she ministered. "As a nurse," she observed, "this is what I can say: The English soldiers —and I have cared for many—have tact, delicacy, and gratitude. But the Boers! No, I pray you, do not let us speak of it." It is only fair to remark that, as mentioned in these columns, some English nurses have found Boer patients extremely grateful for their services.

### NURSING FOR THE MIDDLE CLASSES.

"Shortly after the formation of the Redruth District Nursing Association in October, 1894," writes a Cornish correspondent, "the subject of the district nurses attending middle class patients when not otherwise engaged was discussed by the committee, and it was decided that when not fully occupied in nursing the sick poor there would be no objection to the nurse attending middle-class patients. A clause to this effect was inserted in the minute book, and a table of payments arranged, but except in one or two instances this table of payments has never been required, it having been found by actual experience that middle-class patients availing themselves of the services of the district nurse have gladly paid without waiting for any charge to be made. Indeed, in the majority of cases the amounts sent in have been much in excess of the proposed scale of payments on table. One of the first patients was an official rescued from a wrecked steamboat at a small seaport about three miles from Redruth, in a parish in which no district nurse was available. Although, strictly speaking, this seaport was beyond the confines of Redruth, in the interests of humanity the hon. secretary at once dispatched the nurse to the scene of disaster, and there, in cramped lodgings, Miss Brindley, the nurse, attended the injured patient, with the result that after his return to convalescence the shipping owners sent a cheque for over eight guineas to the nursing fund. Contributions from patients have been received as follows: 1895, £11 7s. 9d.; 1896, £8 12s.; 1897, £2 7s.; 1898, £11 15s. 6d.; 1899, £8 1s. 6d.; 1900, up to this date, £9; total, £51 3s. 9d. Beside this, grateful letters from patients and friends fully testify as to the appreciation of the services of the nurse, which have been, indeed, a boon to the middle-class sick of this district, who but for her help would have fared badly, it being excessively difficult, sometimes even where expense is not an object, to get trained assistance at short notice. The contributions mentioned varied from eight guineas, five pounds, three guineas, down to five shillings, and this by no means represents the total financial benefit, some persons having become regular annual subscribers to the nursing association funds entirely in consequence of beneficial help to the middle-class sick."

### BRADFORD NURSES' INSTITUTION.

The question of dividing the charitable branch from the parent Nurses' Institution at Bradford was discussed at a general meeting of members. It was explained that the institution was founded in 1872 for training private nurses, but that three years later two nurses had been engaged to attend the sick poor. By degrees the number was increased to five, and in 1895 a building for the separate use of district nurses was rented. Recently, it seems to have been felt that there was an element of incongruity in conducting a purely charitable organisation, such as the district nursing branch, in conjunction with the Nurses' Institution, and that it would be to the advantage of all concerned if the district nursing could be continued absolutely independent of the institution. The Board having carefully considered the matter, it was recommended at the meeting that the charitable branch should be launched as a separate organisation. This recommendation being agreed to, a grant of £500 was allocated to the charitable branch, which will henceforth be a separate organisation.

### THE SOUTHGATE VILLAGE NURSE FUND.

It is satisfactory to learn from the fourth annual report of the Southgate Village Nurse Fund that the movement is steadily making progress. In fact, last year was a record year, the number of new subscribers being 30. Another point is the success of the house-to-house collection among the poor, upwards of £5 being obtained in this way, chiefly in pennies. Nurse Thomas, who has been connected with the association for nearly three years, paid 4,626 visits, and owing to an outbreak of diphtheria she had the assistance of two special nurses. The difficulty experienced in securing the latter rooms gives force to the plea that a nurses' home is badly wanted in Southgate. The fête at Southgate House, which was lent by Mr. and Mrs. Feiling for the purpose, resulted in a profit of £105, and of this £100 has been invested. The committee hope to keep it as a nest egg towards the home. We trust that they will speedily increase the size of the nest egg.

### INQUIRIES RE APPLICANTS FOR SITUATIONS.

An animated discussion took place at a meeting of the Ipswich Board of Guardians the other day, in consequence of the production by the Clerk of a telegram from the Master of Sudbury Workhouse addressed to the Master of Ipswich Workhouse, respecting an applicant for the position of nurse on the male side of the infirmary. It appears that the telegram, which suggested that the applicant was not strong enough for the work, was sent under the impression that the post was that of night nurse, and, as the Guardians ultimately selected the lady referred to in the telegram, no harm was done. But a very proper resolution was submitted and carried, to the effect "that all official inquiries, *re* applicants for situations, only be made by the Clerk, except when the Board or one of its committees decided otherwise." The Master of Ipswich Workhouse, whose unauthorised letter to the Master of Sudbury Workhouse resulted in the telegram being sent from the latter will, no doubt, refrain in future from undertaking a duty which does not devolve upon him.

### NURSES' PICNICS.

The nursing staff of the Leeds Union Infirmary have been fortunate enough in having spent two very enjoyable days in the country, the place selected being Boston Spa, by the side of the River Wharfe. The drive, which is nearly 14 miles, was much appreciated, the scenery around Wharfedale being most picturesque. On arriving at Boston Spa the nurses partook of the many good things provided for them by the Guardians, afterwards dispersing in different directions—some to boat on the Wharfe, others to stroll through the woods, and enjoy themselves in various ways, returning laden with flowers to cheer the patients they had left behind. The weather was all that could be desired, with the exception of a slight thunderstorm late in the afternoon of the second day, which, however, did not seem to take much effect upon those on pleasure bent.

### QUEEN'S NURSES AT COLCHESTER.

At the second annual meeting of the Colchester District Nursing Association it was stated that the nurses had paid 4,280 visits. In 36 cases the help of the nurse was enlisted by the doctor, in 28 by the clergy, in 35 by district visitors, and in 44 by the patients or their friends. During the year the committee lost the services of the head nurse, Miss Browne. The balance-sheet showed a balance in hand of £113, and the work of the nurses has been reported upon by one of the inspectors of the Queen Victoria Jubilee Institute, and pronounced "very satisfactory."

### "LIKE YOU, NURSE."

On the evening of one of the numerous carnivals a small boy, aged ten, brought another, aged five, to the casualty ward of a London hospital. "What's the matter, Tommy?" asked the nurse. "Well, nurse, I knows this ere little nipper's mother, I does, and I 'ave just picked him up all covered with dust; you should have seen him, lots of blood on him too. See, there's where it comes from," pointing to a cut on his head, "but 'tis nothing now to what it was, for I have rubbed him down a bit and knocked some of the dust off, 'cause they tell us at the school that dust harbours germs, tho' what germs has to do with harbours I don't know, but teacher tells us, and she knows a lot, she does. This 'ere chap was left at home to mind the baby, but she fell asleep. So he stood at the door, and a young nipper, passing by, called out 'Come and see the carnival.' 'What's that?' said the youngster. 'Oh, just bands and flags, and horses and trumpets and peacocks' feathers.' Off they both ran, and this 'ere chap tried to do a bit of climbing; but bless you, he was not up to that job, and down he fell." A sorry spectacle the youngster looked. On being asked what he saw of the carnival he said, "Ladies dressed like you, mum, running about with boxes with money inside, and they kept a shaking of 'em, they did." He was most stoical while the wound was stitched up. When his mother arrived she nearly pulled his bandage off in the joy of finding him safe and sound.

### ENTERTAINMENT TO BIRMINGHAM NURSES.

On Saturday afternoon upwards of 40 sisters and nurses from the General Hospital, Birmingham, were entertained by Mr. and Mrs. Gilbert Barling at their residence, Edgbaston. Mr. Barling is one of the surgeons of the General Hospital, a member of the Nursing Committee, and always a keen promoter of the welfare of the nurses. A large four-horse omnibus was provided for taking the party to and fro. On arrival refreshments were served in the pretty grounds, and games were soon in full swing, the shooting gallery in the billiard-room being a great attraction and amusement; there was keen competition for the pretty prizes in the final rounds. At tea time the party divided, some going to the drawing-room for a short concert, which was repeated later to the others. The programme was as follows: Pianoforte solo, Miss Haselden; violin solo, Miss Edith Barling; song, Miss Barling; violin solo, Miss Elsie Hopkins; recitation, Miss D. Edmunds. The weather was all that could be desired, and Mr. and Mrs. Barling were untiring in their efforts to give pleasure to their guests. The party left at seven p.m., after spending a very enjoyable afternoon.

### SHORT ITEMS.

Lady Georgiana Curzon has received a cable advising her that the doctors and nurses who sailed in the "Norman" for the branch Imperial Yeomanry Hospital, which is to be established at Pretoria, had arrived all well, and were proceeding to Deelfontein, *en route* for the former town, where they are to be joined by the rest of the staff, who sailed a week later.—At the annual meeting of the Romsey Jubilee Nursing Home it was stated that it had been decided to have a fête to provide funds for properly furnishing the home. Mrs. Montgomery, the retiring president, mentioned that Bacon, the champion runner, had consented to attend and give a five-mile exhibition of running with a bicycle and his pace-maker.—Miss E. M. Byles, the new first assistant matron at East Dulwich Infirmary, was night superintendent and sister at Addenbrooke's Hospital, Cambridge.—The Grocers' Company has made a grant of £50, and the Mercers' Company of £21, to the Hostel of St. Luke, the Clergy Nursing Home, 16, Nottingham Place W.

### Lectures on Medicine to Nurses.

By H. A. LATIMER, M.D. (Dunelm), M.R.C.S. Eng., L.S.A.Lond.; Consulting Surgeon, Swansea Hospital; Past President of the South Wales and Monmouthshire Branch of Brit. Med. Assoc.; Past President of the Swansea Medical Society; Hon. Life Member of the St. John Ambulance Association, &c.

### THE SPECIFIC FEVERS.—INFECTION AND CONTAGION AND MODES OF THEIR OCCURRENCE.

HAVING, in the preceding lectures, dealt with some of the general principles concerning the origin, causes, progress, and results of disease, I now intend to consider special classes and varieties of ailments; and I think I cannot do better as a starting point in this direction than by proceeding in the orthodox manner by giving a description of the definite or specific fevers. These fevers form quite a class to themselves, and inasmuch as they are caused by the operation of germs in the blood and tissues of the sufferers, they are often —especially by the older writers—called zymotic diseases, the word zyme being a Greek one, which means a ferment. It was thought that a specific organism gained access to the blood through some channel, and that, having made good its arrival there, it underwent development and multiplication; that it then produced a disturbance of the whole system after its own peculiar method; and that when it had done this—like in a fermentative process elsewhere—it exhausted the power of the blood to entertain its like again, and so to allow of a repetition of the same illness. And, without a doubt, this theory holds good in the majority of cases of the zymotic diseases, it being a well-known fact that those who have suffered from one attack of small-pox, chicken-pox, scarlatina, whooping cough, enteric fever, mumps, and typhus fever rarely again become affected by the same disease. But still, like everything else in life, this rule has its exceptions, and whilst it is exceedingly rare for repetitions of these diseases to take place in the same subjects, you will every now and then meet with people who have had second attacks of them. I met, for instance, a man some years ago, who had gone through two attacks of small-pox and had been badly marked by them; and I have often seen people who have apparently been rendered immune by a previous illness with scarlatina suffer from exposure to a fresh infection from this complaint, showing it by falling ill with sore throat, feverishness, and even the supervention of the kidney affection, which is one of the marked sequels of that specific fever.

This feature of the zymotic diseases, of one attack of one of the fevers conferring an immunity upon the patient against a repetition of the same ailment is stronger in some—and especially in those I have quoted above—than it is in others of the class. For instance, it is notorious that measles often attacks the same person more than once, and influenza, which clearly belongs to the same order of diseases, seems to go to the other extreme in the matter of exhausting the patient's susceptibility to its attacks, and behaves in such wise that one exhibition of it in a person creates a tendency to recurrence in the same quarter at the next epidemic of the disease. So, although the term zymotic is an excellent one, I think the other names applied to this class of special fevers are perhaps better still. If we call them specific fevers we express very clearly the fact that they are not due to general irritation, but that they have especial, peculiar, and distinct causes; and if, using a still more technical term, we call them the idiopathic fevers, we state the same fact in another way, as the word is derived from two Greek ones (idios—peculiar, and pathos—disease) and so clearly shows that the disease in question is primary in character, and is not consequent upon any other morbid affection. All of them owe their origin to the disturbance set up by distinct varieties of micro-organisms, or germs, which have entered the blood, each germ producing its especial or specific result in the same way that the seed of one kind of plant, when it has fertilised in the ground, produces its like. The view, which you will find held in some ignorant quarters, that dirt and bad drainage will cause the outbreak of scarlatina, measles, or even typhoid fever, is not a correct one. All these specific fevers originate afresh from the operation within the human body of germs which have arrived there from pre-existing attacks of a like disease elsewhere. The lowering of the health of the subject by dwelling in dirty and insanitary surroundings operates only in predisposing him to be attacked when the germ is operating, these conditions generally favouring the development of all low forms of organisms. Being due, as I have just said, to the operation of specific germs in the system, all these special fevers are caused by infection or contagion. These terms are practically synonymous as ordinarily used, yet I would have you draw a distinction between them, as they were undoubtedly to have had originally. The word infection must convey to your mind the communication of a disease by inhaling or swallowing the effluvia of other vehicles which have been charged with the germs set free from one already affected by a like ailment. The word contagion must be held to mean the communication of a disease by contact with one already affected with the same.

From personal observation I should say that very erroneous views are held by the majority of people as to the relative importance of infection and contagion, the last mode of causing disease being placed first in the order of merit, whereas it should really be relegated to quite a subordinate position; for the majority of these specific disorders are due to infection, only very few being caused by contagion. Those caused by direct contact only are the three which I will now name: Hydrophobia or rabies, glanders or farcy, syphilis. You will note that two of these diseases are communicated from animals to human beings. In all of the three diseases the special infective agent has to be introduced into the system through direct contact with a mucous membrane or an abraded skin for the germ to become infective; the dog, the horse, or the human being affected by either of them is harmless to others, provided the contagium be kept within these limits.

How, then, are the other infections introduced into the blood, and through what channels do the infective agents escape from the bodies of people suffering from disease that they may again cause outbreaks of similar complaints in fresh subjects? The answer to these questions varies with the complaint under review, and I will deal with it more fully than I propose to do now when the appropriate occasions arise in the separate review of the various diseases I shall be making. But when I say that the micro-organisms of disease escape from infected people by the breath, skin, and other discharges, and that they effect entries to the bodies of others mainly through the air passages and the alimentary canal, I shall have indicated in a general manner what takes place. But a few words on the general manner of effecting a lodgment in the system will not be amiss.

I take it that it is a general principle in the lives of low organisms that, given a soil on which they can find a lodgment, and a suitable food, they will fertilise with extraordinary rapidity. We are witnesses of this fact ordinarily in everyday life when we see a field of wheat or a crop of potatos quickly covered with "blight," and when we hear of vineyards being spoilt by phyloxera. So it is with the special organisms which cause specific diseases. They may be compared to the low-formed organisms I have been speaking of, as far as their sudden appearance and their rapid development is concerned. Gaining access to the blood by one of the channels I have indicated, they quickly develop there and produce the definite symptoms of the diseases they are the conveyors of. A few considerations will show us by what quick and easy methods they can do this, and these I will proceed with in my next lecture.

### The Hospital Ship "Princess of Wales."

### INTERVIEW WITH THE ACTING SUPERINTENDENT, A.N.S.

By a Special Correspondent.

"I wouldn't nurse civilians if I could help it," said Sister Chadwick emphatically, "for anything. I consider Tommy the best patient in the world, and very grateful for anything you do for him."

"You have been army nursing for several years, have you not?" I asked.

"Nearly fourteen years. I have been at Netley, Woolwich, Malta, Gosport, and Colchester, where I went as acting superintendent. I went on from Colchester as acting superintendent on the Hospital Ship 'Princess of Wales.'"

I asked Miss Chadwick to tell me how she got this important post, and she told me that when the war broke out she was simply mad to go to the front. She hoped to be appointed superintendent of a hospital ship, as she was such a good sailor, and asked if it was possible for her to be appointed superintendent of the "Maine," but found that it had been decided to appoint an American lady.

"I returned to Colchester," said Sister Chadwick, "in despair; but the following day a telegram came down to say that I had been appointed acting superintendent of the Hospital Ship 'Princess of Wales,' and that I was to hold myself in readiness to embark immediately."

"Do you never suffer from sea-sickness?" I asked.

"No. The ship may stand on her head, and I don't care. The only thing is, I am rather shaky on my legs, after the fever I had in Malta, and I can't walk straight if the sea is a bit rough."

### The Floating Hospital.

Sister Chadwick was enthusiastic about the hospital ship, with its modern appliances.

"One special thing to remark is," she said, "the headroom we have. And the ventilation I consider excellent, as the wards, even in the Tropics, were never stuffy. Then there were sea baths for the patients, and these they much enjoyed. The electric light, and above all, an electric kettle for making tea at all hours, which we had in our quarters, were invaluable. Also the electric irons, which we could use ourselves for ironing aprons; and I even pressed some of the men's khaki clothing before we got to Southampton. It is without exception the finest vessel I have seen, from a nursing point of view. There are separate cabins for the sisters, and my own room is simply charming. It is the shape of the letter L, and I can draw a curtain across the inner part and be quite private. We can get on deck in two minutes in case of emergency. Each sister has a nice little room attached to her ward, for keeping medicine, dressings, lotions, &c., as no poisons, of course, are ever allowed in the wards. The operating theatre is a very nice one, fitted up with the *x* Rays."

"How many sisters are there on board?" I asked.

"Myself, as Acting Superintendent, and three Army Nursing Reserve Sisters, Sisters Spooner, Hogarth, and Brebner."

"And medical officers?"

"The principal medical officer, retired R.A.M.C., and Adjutant R.A.M.C., and three civilian doctors."

"And you have had the same sisters throughout?"

"Yes, and I wouldn't change them on any account; besides being excellent as nurses they are personal friends."

"How many beds are there in a ward?"

"Forty on an average. The smallest ward has thirty beds, and there is a small isolation ward attached for serious cases. Most of our beds are fixed, but some are swinging cots; the men prefer the fixed ones. It is wonderful how fond they get of the ship. There was one man who came on board at East London and had to be landed at Cape Town, as he was to join Greenpoint Camp. Well, when we arrived, the roll was called, and he didn't appear. His name was shouted all over the ship, and at last he was found. He had been hiding, hoping we should leave for England and bring him with us."

### The Visit of the Princess.

"Another man," Sister Chadwick went on, "had a very bad wound caused by an expanding bullet, and his leg had been amputated. He was very anxious to stay with us and make the voyage to the Cape again. The Princess of Wales heard of it, and asked whether we couldn't take him back. But of course this was impossible."

"You had a visit from the Princess of Wales the other day, had you not?" I inquired.

"Yes; there was a rumour that she was not coming, and I can't describe how disappointed we all were. But when we knew it was a mistake, and that she was really coming on Monday, the greatest excitement prevailed. We were all charmed with her visit; she asked so graciously after our accommodation on board, and even inquired if our beds were comfortable. She went through all the wards, and spoke to every man on the ship; and she expressed herself highly satisfied with all she saw. We had the honour of accompanying her to the train."

### The Spoiling of "Tommy."

"You are not a Red Cross sister; but was not the ship built for the Red Cross?"

"No, chartered, and I am lent to her. I am an Acting Superintendent Army Nursing Service, and I wouldn't change for worlds. My 'lentness' only applies to my appointment to the ship."

"Then," I said, "as you are in the Army Nursing Service, of course you stand up for the War Office, and disapprove of the complaints now being made?"

"Most certainly. Civilians can't understand the exigencies of war; and as for the British public, I have no patience with it! They expect everything to be done in the wilds of Africa as if they were in the middle of Hyde Park, with everything at hand, and they talk about what they don't understand. It is impossible to work a military hospital on the same plan as a civil. Think of all that has to go up on one single line, with narrow gauge, to Pretoria —troops, ammunition, guns, food, transports, horses. My own opinion is that all this spoiling of Tommy is extremely bad for him; it is being overdone, and will be the ruin of the discipline of the army if we don't take care."

"Still," I remarked, "he has suffered terribly during the war."

"Yes, I quite agree. It made my heart ache to see the men when they came on board, like skeletons, the skin just drawn over the bones. They said it was many months since they had seen such a comfortable place as the wards. They should have every consideration; but it is just as bad to overdo it as it is to underdo it."

### The Nursing.

"And now," I said, "will you tell me how the nursing is done?"

"We have a Sister in charge of each ward, who is assisted by a wardmaster for discipline, and orderlies who carry out instructions given them by the Sisters, who of course receive their orders from the Medical Officer in charge of the ward. Civilian nurses have an idea that an army nurse has nothing to do, but I have heard them confess that when they have joined us it has been an 'eye-opener' to them. The bulk of the work is done in the morning and evening. I call it a sign of bad nursing to leave a lot of work for the afternoon. The sisters take it in turns to do night duty, and we take afternoon duty in turns. One sister is always available at a momen t's notice. My wardmaster and orderlies were invaluable. They helped me in every possible way they could, and they had long hours of duty."

I asked Sister Chadwick how long her leave lasted, and when the ship was to start again.

"I hope next week," she replied. "This is not 'leave,' but 'enforced absence from duty.' The ship is in dry dock, so we can't be on board; but I am longing to go back."

"There is not much to do on the voyage out, I suppose?"

"No nursing, of course, unless any of the crew or staff get ill, but all the parcels addressed to me I have to open, and then all the people who have sent things have to be written to and thanked."

"I daresay you get a great many delicacies for the patients?"

Miss Chadwick laughed. "Yes," she said. "As I told you, Tommy is being completely spoilt. But, of course, it's better to have too much to give him than too little, though it's almost as difficult to know how to divide the things."

### One Touch of Nature.

Sister Chadwick is devoted to animals and birds, and thinks them "infinitely preferable to humans." It is her one trial that army orders forbid the keeping of pets on board ship.

### A Function of the Week.

### LONDON HOSPITAL.—OPENING OF NEW CLUBS UNION.

#### Distribution of Prizes to Nurses.

On Thursday last week a combination of most interesting functions took place at the London Hospital, when Mr. Treves, consulting surgeon to the hospital, opened the New Clubs Union's rooms, gardens, and fives court, and also distributed prizes to the students and nursing probationers.

Amongst the company who assembled in the library of the Medical College to meet Mr. Treves were Mr. Douro Hoare (chairman of the college board), the Hon. Sydney Holland (chairman of the hospital), Dr. Stephen Mackenzie (senior physician of the hospital), Sir Frederick Young, Sir Edmund Hay Currie, Lady Lucy Hicks-Beach and Miss Hicks-Beach, and many others, including members of the House Committee and the hospital staff, medical and surgical.

The visitors first inspected the new rooms, the garden, and the fives court, all of them most delightfully arranged for the comfort and pleasure of the medical students. The rooms are ample in size and most admirably furnished. Their cost has been defrayed by subscriptions from members of the House Committee and staff. The garden has been well terraced and laid out, and is indeed a place of rest and refreshment; whilst the Eton fives court (the gift of Mr. T. Fowell Buxton) is ready to be used for recreation and exercise.

The party next returned to the library, when Mr. Douro Hoare briefly opened the proceedings with a reference to the great services performed by Mr. Treves in South Africa.

Mr. Treves, who was received with prolonged cheering, said he felt honoured by his reception, and by being asked to present the prizes. He himself had not won prizes in his student days; in fact, he had been given Hogarth's pictures representing the idle and industrious apprentices—as a solemn warning to himself. Success depended largely upon oneself, he thought. There was no such thing as luck. A chance would come, and out of fifty men only one would seize that chance. He considered genius a species of neurosis, and persons of genius impossible people. Hard work was one faculty needed in the medical profession, combined with perfect honesty and much kindness, and above all, the power of close observation. He also referred to the changes in hospital administration in the last twenty to thirty years—changes greatly to the advantage of the students. He finally referred to his work in South Africa, and mentioned that with the Moveable Hospital No. 4 they had four nurses, the only ones who were out within the actual range of fire. Two of these were "London" nurses—Nurse Tor and Nurse Salmon.

The prizes and certificates were then given to the students, Mr. Munro Scott (warden of the college) reading out the names. In Mr. Scott's speech he laughingly remarked that he had some hesitation in asking Mr. Treves to give prizes to women. But he thought that women who had gone through the mill of training in a London hospital were worthy.

Mr. Sydney Holland proposed a vote of thanks to Mr. Treves. He then observed that out of 104 nurses who went in for examination 103 passed, only one failing on account of illness. He spoke of the installation of the "light cure" in the hospital, and of the interest taken in it by the Princess of Wales, who had installed it. He also mentioned the nurses who had been sent to South Africa at the request of the Princess, and made a sympathetic reference to Nurse Evans, who had died at the seat of war.

Mr. Treves, in distributing the prizes to the probationers, mentioned that the first prize, gained by Miss Ethel Canty, was a cheque enclosed in one of the Queen's chocolate boxes, found in a trunk outside Ladysmith. The second and third prizes were gained respectively by Miss M. M. Southcott and Miss G. Yates Grant.

Dr. Stephen Mackenzie, in seconding the vote of thanks to Mr. Treves, spoke of him as the man of the day, and alluded warmly to his South African work.

The hospital was thrown open to visitors, and the Lawler Orchestral Band performed a delightful selection of music in the garden, where refreshments were served. The nurses in their cool frocks, the students in their best coats, and the smartly-clad visitors, made up a pleasant picture, and offered a strange contrast to the bustling world outside in the Mile End Road.

### Presentations.

Ramsbottom Cottage Hospital.—Miss Wareing, who has been appointed to a post at the London Hospital, has been presented with a handsome dressing case and purse of gold containing £20 from a number of friends in recognition of her services, extending over a period of six years. She rendered valuable help in the building up of Ramsbottom Cottage Hospital, and it was owing to her successful work that the donors were led to think of establishing it.

Fir Vale Infirmary, Sheffield.—Miss R. M. P. Rochford, on resigning her post as night superintendent of nurses to take up private nursing in London, was presented with a very handsome case of brushes, &c., beautifully mounted with silver, by the medical and nursing staff as a token of their esteem and good wishes for her future welfare and success.

Leeds Union Infirmary.—Nurse Goodall, who was trained for three years at Leeds Union Infirmary, and promoted to the post of charge nurse on the completion of her training, was presented with a silver sugar basin, tongs, and cream jug by the officers and members of the nursing staff on the occasion of her leaving to be married.

### Compulsory Registration of Midwives.

ANNUAL MEETING.

THE Association for Compulsory Registration of Midwives held its annual meeting, by kind permission of Lady Lucy Hicks-Beach, at 11, Downing Street. There was a very good attendance, and the discussion on the Bill was somewhat lively. People have been known who "came to scoff and remained to pray," and it was noticed that the lady who rose to protest *in toto* against the Bill afterwards joined the association.

THE MIDWIVES BILL.

Mr. HEYWOOD JOHNSTONE, M.P., was in the chair. In his opening remarks he reviewed the history of the Bill. At the beginning of March, he said, the cause might have been given up as hopeless; he had almost tied up his papers and dismissed the matter from his mind for another year. But suddenly one afternoon there had been an opportunity of getting the opinion of the House of Commons, and although the matter had not been brought to a conclusion, he had been well satisfied with the discussion as far as it went, and there was reason to hope that substantial progress had been made. It was very disappointing that there was now no prospect of its coming again before the House this session, but he thought that could hardly be expected for a private member's Bill. It was very easy to obstruct a Bill, and those who sought for opportunity to do so could readily find it, just as it did not require much brain on the part of a little vulgar, ignorant boy, when he had made up his mind to send a train off the rails—a stone or a beam would serve—so it was not difficult to raise enough talk to take up the time of the House of Commons, and effectually prevent or delay the passage of a useful and desirable measure. The objections that had been raised he described as "ill-founded, illogical, and hopelessly and irredeemably irreconcilable." But the discussion had elicited the opinions of three medical M.P.'s, of whom one approved of the Bill, one gave no certain sound, and a third said it ought to be taken up by Government.

TRAINING AND SUPERVISION.

The Chairman alluded to the travellers on the road to Jericho, when a certain man fell among thieves; he thought probably ninety per cent. of the passers by said, "Government ought to do something." It was of no use waiting for Government; we had to create the interest, and educate our masters as to the need, and the way in which the need is to be dealt with. He had waited on Mr. Balfour with an exceedingly important memorial; he had never seen a more weighty document, or one signed by persons more qualified to give an opinion, and he hoped that the Government would prove themselves alive to its importance. "Let us," he concluded, "leave details, in a sense, to those objectors who contradict each other, and keep our minds fixed clearly on the two principles of the Bill—the need for training and supervision." Delay and defeat had repeatedly, in history, proved to be the right road to success, and he hoped that the promoters of this Bill would add another instance to the number.

"THE FRIENDLY NEIGHBOUR."

Mrs. Wallace Bruce, chairman of the Executive Council, explained what she had found from experience to be a difficulty with many people, who imagined that, when the Bill was passed, a neighbour going in to help a friend in time of emergency would be liable to a fine of £5. The framers of the Bill had guarded against this possible interpretation by inserting the word "habitually"; she became liable to the penalty if it was made a practice of for gain.

A lady who inquired whether uncertificated midwifes who had been practising for many years would be liable in the same way, received the reply that this also was provided for in the Bill; such midwives, on production of testimony to their respectability, and on showing that they had been practising for many years, would be licensed.

THE RESOLUTION TO THE LORD PRESIDENT OF THE COUNCIL.

The following was the resolution before the meeting:—

"That this meeting begs very respectfully to draw the attention of his Grace the Lord President of the Council to the fact that, as the Government have now taken the whole time of the House of Commons for the remainder of the session, the Midwives Bill cannot be further proceeded with, although its second reading was carried by a large majority, and it passed through Grand Committee in a form substantially acceptable to the General Medical Council. Having regard to the admitted need for legislation on this subject, and to the opinions expressed by members representing the medical profession in the course of the adjourned debate on June 27th, this meeting would appeal to his Grace to bring the matter before the Cabinet with a view to its being included in the Government legislation of next session."

It was moved by Dr. PERCY BOULTON, who said he remembered the time when about ten women presented themselves for examination in widwifery. Now the class of woman taking up the work had been entirely transformed, and many of them passed as well as an average student. The candidates numbered over 1,000 yearly, and it took ten examiners, giving two evenings' hard work four times a year, to get through the work. He thought very few voluntary bodies could do the work of the Obstetrical Society, and it was time that the matter was taken up by Government.

THE HUNDREDTH CASE.

In seconding, Dr. BOXALL drew a sharp distinction between a midwife and a nurse; the latter always acted under the supervision of a doctor, the former did not always do so. The distinction, which people would not bear in mind, was a very important one. This country was considered behind the times; it certainly was so in a matter like this. It devolved on the midwife to make certain examinations of the patient. Puerperal fever was a great scourge, the death-rate from it was enormous. It could be avoided, but only by carrying out directions. People imagined that experience taught a midwife her duties. "It doesn't matter how many babies she may have successfully brought into the world," said the speaker, "she can still carry infection. No amount of seeing babies born—by the hundred, if you like—can teach her this." Ninety-nine cases might be normal; but a midwife must be able to foresee, and able to deal with the hundredth case which was not normal, and the use of both doctor and midwife was for the hundredth case.

ON BEHALF OF THE MIDWIVES' INSTITUTE.

Miss ROSALIND PAGET, speaking as a practical midwife, said that the present Bill was not, as some people thought, promoted by the Incorporated Association of Midwives. The Bill of 1890 was, and, personally, she preferred that one. But her association had agreed that any Bill was better than none at all. The poor mother must come first, then the midwife, and lastly, she thought, the medical profession. Her association gladly welcomed supervision.

### To Nurses.

WE invite contributions from any of our readers, and shall be glad to pay for "Notes on News from the Nursing World," or for articles describing nursing experiences, or dealing with any nursing question from an original point of view. The minimum payment for contributions is 5s., but we welcome interesting contributions of a column, or a page, in length. It may be added that notices of entertainments, presentations, and deaths are not paid for, but, of course, we are always glad to receive them. All rejected manuscripts are returned in due course, and all payments for manuscripts used are made as early as possible at the beginning of each quarter.

### The Nurses of Kent and Canterbury Hospital.

### A CHAT WITH THE MATRON.

By Our Commissioner.

Of course, the Cathedral dominates Canterbury. Its antiquity, its traditions, its splendour, invest it with surpassing interest. The ordinary visitor probably finds nothing else in the city worth seeing, and pays little heed to an institution adjacent to the historic edifice which provides for the wants of the sick and suffering poor in the "Garden of England." If the stately Cathedral is the centre of religious worship, the Kent and Canterbury Hospital is a perpetual witness to the practical character of Christianity. Of unpretentious architectural dimensions, it stands well back from the roadway, and has grounds which, if not large, afford that "touch of the country" so keenly appreciated alike by patients and by nurses.

The matron was good enough to show me over the hospital immediately upon my arrival, and I was therefore able to form some idea of the work of the nursing staff before I asked her any questions. I also inspected the nurses' home, which is excellently arranged and appointed. The distance from the hospital is inconsiderable, and pavement is laid down, but some further protection from the rain would be an advantage. Miss Messum, who was trained at St. Thomas's, and had charge of Sir William MacCormac's male surgical ward, would be only too glad if this and other improvements on modern lines could be effected. But, as she explained, the hospital is greatly crippled for want of funds. When it was erected, more than a hundred years ago, its claims were generally recognised by the county, but there are now several hospitals in Kent which appeal for support, and fresh subscriptions are hard to obtain.

### The System of Training.

"But at the same time," said the Matron, "we have not stood still."

"I suppose you have seen important changes?"

"The most important during the nine years I have been here has been the alteration in the system of training. Until I came the majority of nurses were trained for one year only for the Canterbury Nursing Institute. The Institute readily altered the training to two years on my application. A fair and increasing number apply to stay for three years, but two are compulsory, except in the case of a few probationers who come for one year and pay a premium of £20. Most of the latter ask to remain for the second year."

"Do you regard two years as sufficient?"

"I do not consider that two or even three years in a hospital like this qualifies for the higher posts, or where a certain amount of power is needed. But I do think that two years' training should be sufficient for district work. Virtually at the end of a year a woman is made a charge nurse in most hospitals, and she becomes at once responsible. In my opinion, the women trained in small country hospitals often prove a safe and reliable corps in the nursing world, and make admirable nurses for private or district work. They take their own place, and are as necessary to the profession as the country hospitals themselves."

### The Value of Small Institutions.

"In a small institution in a quiet city like Canterbury, numbering about a hundred beds," continued the matron, "with no medical school attached, having chiefly chronic cases, and major operations few and far between, the conditions under which the probationer is trained differ widely from the large established nursing schools of London and other great cities. So it falls out that much less is required all round. I have had several probationers who failed to 'get through' in a London hospital, but who did well here, and in the positions they afterwards obtained. But I am always glad when the probationer who has been here two or three years joins a large hospital as staff nurse, and thus widens her experience."

"Does that often occur?"

"Frequently. There are no appointments here for the nurses after they have finished their training, and unless they join a large hospital as staff nurses, they devote themselves to private or district work."

### Hours and Holidays.

"What are the hours of duty?"

"Sisters are on duty from 8 a.m. until 9.15, with off duty on alternate days, either afternoon or evening, half a day a week, and a day a month. They are allowed a holiday of four weeks annually. Probationers are on duty from 7 a.m. until 8.30, and are off duty alternate days two hours in the morning or three in the afternoon, and a day a month. They have a week at the end of the first six months, which I think they greatly need, and three weeks' holiday at the end of the first year of probation."

"How about instruction?"

"We attach great importance to instruction. Three of the visiting staff give lectures regularly during nine months of the year on anatomy, physiology, and hygiene, and I have classes four times a week in the winter and twice in the summer. A very thorough examination is made by one of the visiting staff at the close of his lectures."

"At what age are probationers admitted?"

"From 22 to 35. I think 22 is quite young enough, and, on the other hand, it is difficult for the average man or woman to assimilate after the age of 35. I am opposed to an age limit for retirement. Women are often at their best between 45 and 60. Their organising power develops."

### Importance of the Laundry.

"Has the staff been increased in your time?"

"Yes, from 13 to 22. Four are sisters, and 18 probationers in different stages of training. There are 106 beds, divided into three floors, male and female, medical and surgical. There were no bath-rooms for the wards when I came, and we have lately introduced more convenient and comfortable baths, as well as improved sanitary arrangements generally."

"I see that you have a drying-room for the laundry."

"I should like to emphasise the importance of the laundry which is attached to the hospital. There is more complete supervision, and ability to prevent the clothes of the staff from being mixed with those of the patients. This is impossible where there is no drying-room. There is also a drying-closet in the Home, and nurses are told not to use their clothes until they have been in it."

### The Nursing Home.

"When was the Nursing Home built?"

"Three and a-half years ago, at a cost of about £1,500. As you said just now, there are good-sized dining and sitting rooms, eleven bed-rooms, and other requisite accommodation. Both in the Home and in the hospital there are double-bedded rooms as well as single. The tendency is, I know, to go in for all single rooms, and it is very nice for a nurse to have a room to herself. So far as sentiment goes, the single room may be better, but on health grounds, where space is limited, I favour the larger double apartments, as being more airy. The sisters have a bed sitting-room of good size, off the wards. I should like them to have their sitting-room off the ward and their bed-room in the Home."

"But I apprehend that the question of cost comes in?"

"Unfortunately it does. It is only because we cannot afford it that the way between the hospital and the home is not covered. I do not think, however, that nurses who provide themselves with goloshes run any risk of injuring their health, assuming that they are fairly strong; and unless a woman is fairly strong—well, she should not be a nurse."

"Are you often in the home?"

"I take all my meals with the nurses, and have always done so."

RECREATION AND THE SOCIAL QUESTION.

"Tennis, I notice, is a recreation."

"Both tennis and croquet are provided, but under the new regulations of the latter game the nurses do not seem to care about it. Many of them cycle, and go off for a day as far as Herne Bay. There is a piano in the room."

"And a theatre in the City?"

"Yes; but I very much object to the probationers going to the theatre here at night. I am also strongly opposed to dances in hospitals. The committee are of the same opinion in respect to them, and we therefore never have any dancing. I think, however, that representations at Christmas in the wards often afford much pleasure to the patients, and, providing that they take place where there are no serious cases, can see no reason why they should not be given."

"You do not, I gather, object to the theatre *in toto?*"

"No, I was only alluding to the young nurses. Those who are trained must decide for themselves. The question of social life for probationers is a very difficult one, and I have come to the conclusion that it is better to leave them to make their own arrangements. I follow this rule with regard to religion. Ample opportunity is given to all nurses to attend which church or chapel they prefer, and I always do my very best to meet the wishes of those who desire to attend the early service."

"Do you care to say anything about pensions?"

"The funds will not allow the committee to do anything, but I am a warm supporter of the Royal National Pension Fund, and I wish that all the nurses here would belong to it."

PRACTICAL PROOF OF CO-OPERATION.

The matron had commenced telling me how ready the nurses are to help each other, when it was announced that a case of an abdominal operation had just come in, and that both the theatre nurse and the sister of the ward were out. But a surgical nurse at once volunteered to take it, and there was not a moment's delay. The spirit of cheerful co-operation and loyalty which seems to pervade the hospital is one of its most pleasing features.

### Nursing at Maritzburg.

NOTES BY AN ALEXANDRA NURSE.

OUR correspondent, who dates her letter June 30th, writes: After seven months uninterrupted hard work I succumbed to an attack of influenza and laryngitis, and have come to Durban for a short rest and change. Some twenty of the men had bad throats and influenza colds, also several of my women patients, so I presume I contracted it while I was nursing them. For the past three months the surgical work has not been so much in evidence at the inspection room, but we have had many patients suffering from pneumonia, acute rheumatism, and chest and throat troubles.

It seemed as if these came in batches and by rotation. First we had a run of men suffering from deafness and discharging ears, swelling and pain being a usual accompaniment. No doubt the noise of battle was accountable for much of this deafness. These cases took a great deal of time, as besides syringing and cleaning carefully, poulticing and fomentations had often to be resorted to. After a short treatment many regained their hearing; others, however, were unfitted for further service through their deafness, and were sent home. Quite a number followed recovering from pneumonia; these had been in hospitals up country or on board the hospital and transport ships.

THE VALUE OF TRAINING IN MASSAGE.

The rheumaticky patients claimed a lot of sympathy, and for these my training in massage was most useful. So many had swollen and stiffened knee-joints and elbow-joints, shoulders and backs not escaping.

One or two of the first, having been relieved by gentle friction, must have passed it on to the others, for I was often reminded by, "It's a grand thing is that massage, Sister; it's a queer power o' good, it is." An application of iodine and a good flannel bandage after, sent them away comfortable if not cured. It was not surprising that a considerable proportion suffered from the throat and chest. The latter showed what the confinement had done for them at Ladysmith. A single application of liniment was in their case sufficient to produce blisters, so easily ruffled was the skin, and so delicate that it reminded me of wet tissue paper.

The tonsilitis cases afforded amusement to the onlookers, for the men, when asked to open their mouths wide and to say "Ah!" in order to have their throats painted, seemed to lose their courage. One or two looked as if they would rather face a Mauser bullet. A photographer could have had some amusing snapshots.

ATTENTIONS TO OUT-PATIENTS.

The ladies of St. Anne's College were most kind in not only giving another equally successful garden party and concert to my out-patients, but they also supplied chest protectors, flannel belts, pneumonia jackets, &c., and sent twice weekly large quantities of sweet or tree tomatos to beguile the tedium of waiting to be dressed. This was no small undertaking, seeing that we had from 80 to 100 patients each morning. Such kindness I accepted as a personal one, as all were more or less invalided, and being out-patients had not the comforts and luxuries of the hospital wards.

On the Queen's birthday, 75 of these were by the invitation of a few friends in town entertained to tea and games in the park, pipes, tobacco, cigarettes, matches, and sweets being most liberally supplied.

A SAD CASE.

One case, that of a young sergeant of the Lancasters, was very sad. He came down from Colenso with a deep and ugly shoulder wound. It improved nicely, and he would soon have been marked fit for duty again. But on the tenth day I found he had fever, and sent him into hospital at once. For the first week all went well, and pointed to a good recovery; but visiting him on the Monday I was disappointed to find that a change had taken place, perforation followed, and he died on Tuesday. I think one of the saddest duties of our profession is when we have to write to parents and friends of the death of their loved ones; and yet I know that they are truly grateful for every word we can tell them of their last hours.

It has been a source of much consolation that my women and children have kept so free from serious illness while the husbands and fathers have been at the front. I had a hasty summons at midnight to a little boy suffering from croup. After an hour's work and anxiety my efforts were successful, and I left him relieved and sleeping quietly. Two more little strangers have come to gladden the home, in the father's absence; one parent is at the front while the other has been in hospital for almost three months with fever.

### The Principles of Antiseptic Surgery and the Preparation of Patients for Operation.

Abstract of a Lecture to Nurses given at the City Orthopædic Hospital, June 19th, 1900, by J. JACKSON CLARKE, M.B.Lond., F.R.C.S., Surgeon to Out-patients at the North-West London and City Orthopædic Hospitals.

AT the last lecture we left the patient prepared for receiving the anæsthetic. We must now briefly consider the preparation of the instruments and the dressing and the conduct of the operation. No human responsibility could well be more serious than that of all who are taking a part, however slight it may appear, in any surgical operation. Any flaw in a long series of precautions may endanger the loss of limb or life; thus, if it should be omitted to open the apertures in the cylinder containing the dressings before it is placed in the steam steriliser, the dressings will not be properly sterilised, and wound infection, with all its dire possibilities, may ensue. In every hospital one half day in each week should be set aside for the inspection of the operating theatre and the ward dressing-boxes. The instruments, sutures, ligatures, stock lotions, dressings, &c., should be inspected; if any are found to be running short they should be made up from the store, and the 1-20 carbolic lotion in which the ligatures, drainage tubes, &c., are kept should be changed and renewed.

The word sterilisation has now become familiar to you all. In surgical work it is effected either by steam or boiling soda solution or by antiseptics. It will serve to give definiteness to our ideas of steam sterilisation if we consider for a moment some of the methods of sterilisation used in bacteriology. In order to provide sterile broth or jelly for the cultivation of different kinds of bacteria, the bacteriologist uses small quantities of these nutrient media in test-tubes plugged with cotton wool. The plugged tubes are placed in open wire-work cages, and so transferred to the heated steamer for over 15 minutes on each of three successive days. In a proper steamer—and during the three years that I was in charge of the bacteriological laboratory of St. Mary's Hospital I found an ordinary potato-steamer answer as well as the more expensive apparatus—the substances are thus kept at the temperature of boiling water for the time that they are in the steamer. The reason that they are steamed on three successive days is because many bacteria form spores that are far more resistant to heat than the bacteria themselves, and these spores, if present in the nutrient media, change to bacteria before the third day, and so are killed at the second or third boiling. Happily, the common organisms that cause suppuration, staphylo and strepto-cocci are readily killed by steam. Some wound-infecting organisms, *e.g.*, the tetanus bacillus, and the bacillus that causes emphysematous gangrene, and possibly the tubercle bacillus or its spores are difficult to kill, and in the steamings of the dressings we must remember this, and to make up for the want of the successive steaming we must give them a longer stay in the steriliser. The dressings should remain at least one hour in the steriliser after the water has begun to boil, as shown by the thermometer.

### THE PREPARATION OF THE DRESSINGS AND OF THE INSTRUMENTS.

The dressings are various, according to the experience of the surgeon. For my part, in clean wounds I employ next to the wound a few layers of the pink gauze which is impregnated with the double cyanide of mercury and potassium. In most cases it adds to the comfort of the patient if the layers placed next to the wound are wrung out of 1-40 carbolic lotion. The anæsthetic property of the carbolic diminishes the smarting of the wound. Over the layer of gauze an ample covering of sterilised absorbent wool is placed, and over this another layer of cyanide gauze to prevent the ingress of bacteria from the outside. The dressing and the protecting towels, which should be unfolded, are placed in the cylindrical receiver of the Schimmelbusch steriliser in the following order: (1) The bandages; (2) the lint or other swabs; (3) the outer layer of gauze; (4) the absorbent wool in thin flat layers; (5) the inner layer of gauze; (6) the protecting towels unfolded and lightly packed in. The apertures in the cylinder are then fully opened, and it is placed in the wire chamber of the steriliser.* The instruments should be boiled for ten minutes in a 1 per cent. solution of crystalline washing soda. A level teaspoonful to a pint of water is, roughly, the right proportion. After this the instruments are placed in 1-20 carbolic lotion, to which, just before the operation, hot water is added to make the lotion a 1-30 one. I prefer not to have the scalpels and tenotomes boiled, but instead to have them well washed with ether soap, and then placed in 1-20 carbolic.

Silk for sutures should be wound on glass spools and boiled for 15 minutes in 1-20 carbolic, and kept in lotion of the same strength.

Chromicised gut, drainage tubes, silkworm gut, and horse hair should be kept in 1-20 carbolic for at least a week before being used.

When the patient is anæsthetised, and the surgeon is ready to begin the operation, the bandages, cotton wool, and waterproof covering are removed, but the lint, moist with 1-40 carbolic, is left in place for the surgeon or one of his assistants to remove at the last moment. If a nurse should be required to thread needles, or otherwise assist directly in the operation, her hands must be as carefully prepared as those of the surgeon, and she should avoid touching any unsterilised object, and if by chance such contact has occurred, she should carefully go through the process of carbolising her hands again before resuming her special task of threading needles or handing sutures.

After the lint dressing has been removed from the part to be operated on, the sterilised towels are removed from the cylinder, and are placed so that their edges lie on prepared skin, and the rest of them cover the adjacent parts of the patient, &c., in order to protect the surgeon's hands, the instruments, and dressings from contact with any unsterilised thing.

During the operation all unnecessary moving about in the theatre should be avoided. If the surgeon requires the dressings to be handed to him this should be done by means of sterilised forceps, and not by the nurse's fingers. For my own part I reserve iodoform for conditions in which infection, whether tuberculous or suppurative, is already present, or when the part cannot be kept aseptic as in operations about the anus. In most cases I prefer to have the iodoform as an emulsion of ʒj to ℥j in glycerine. The bottle in which this is kept should be provided with a glass rod for stirring. Iodoform gauze should contain some oil of green mint in order to mask the offensive smell of the drug. I found that anthrax bacilli grew freely in jelly that contained 1 per cent. of iodoform. The great value of iodoform consists in its property of liberating iodine in infected wounds. The iodine combines with the poisonous products of the bacteria, rendering them inert, and also it tends to diminish the growth of bacteria in wounds.

In the case of children it is often a matter of difficulty to prevent the dressing becoming soiled after the operation. In such cases a covering of jaconet, carefully fixed to the skin at its edges by means of some Mead's strapping, will be of use. Throughout the course of healing as well as during the operation the principles of antiseptic surgery must be kept in mind, and if in spite of careful nursing the dressing should become soaked with urine or otherwise soiled, immediate warning should be given to the surgeon. If blood or other discharge should appear through the dressing, a covering of sal-alembroth or other antiseptic wool should be placed over the part of the dressing where the discharge made its appearance, and the surgeon should be informed of the event without any delay.

* These proceedings were demonstrated to the audience.

### Nursing in Sanitoria for Consumption.

By JANE H. WALKER, M.D.Brux., Physician to the New Hospital for Women, and Medical Superintendent of the East Anglian Sanatorium.

### II.—A DAY IN A SANATORIUM FROM THE NURSING POINT OF VIEW.

A NURSE gets up at about seven, has her breakfast as soon as she is dressed, and then is ready to take breakfasts at eight to all the patients who are in bed. The question of food at a sanatorium for consumptives is an all important one, and cannot be too strongly insisted on. The patient has been seen by the doctor before breakfast, and any special diets or amounts of food regulated. Whatever has been decided on must be faithfully carried out, and it is an absolute rule that no plate must be taken away from a patient till all food on it has been eaten. The nurse's business is to see that this rule is enforced. If for some reason the patient cannot finish his meal then the matter must be referred to the doctor. This rule applies equally, of course, to dinner and to supper. There are ways and ways of taking meals to patients. In the first place the tray should be big enough to contain all requirements, and the tray-cloth should be absolutely clean; there should be no such thing at dinner as a cloth stained with the coffee of the previous meal, nor at breakfast traces of the gravy of the night before. This may seem self-evident, and it may appear unnecessany to draw attention to such a trifling detail, but it is a distinctly important point, and I know, from personal experience, is one frequently overlooked. Next see that the patient is comfortably settled in bed, at an angle convenient not to the nurse, but to the patient (here I know that I am treading on delicate ground, for if a nurse knows anything she knows how to smooth the ruffled pillow and cool the fevered brow, so to speak). Then see that the patient has everything he wants; salt is not infrequently omitted. Remember, too, that some people take sugar with porridge, and others take it with tea and coffee. In fact, a nurse should put herself as far as possible in the patient's place. In other words, she should be really sympathetic without being soft and mawkish.

### AFTER BREAKFAST.

When the breakfasts have been given, the rooms of those patients who are up and at breakfast should be seen to. If it is in winter, and the windows have been shut for the patients to dress more warmly, they must be of course opened at once, and if the heating apparatus has been on it can be turned off. The bed must be stripped and the slops emptied by the housemaids, who should also take up the strip of carpet by the bed and put it outside the room, and the floor must be swabbed with water every morning. The beds should be left unmade as long as possible, and when they are made patients' idiosyncrasies in the matter of the disposal of their bedclothes and pillows should be attended to and marked.

### THE IMPORTANCE OF A WET DUSTER.

When the floor is swabbed and the bed made, then the nurse can do the dusting, which is done with a wet duster. I say wet advisedly; I used to say damp, but experience has taught me that a damp duster is apt to become dry, and so defeat its own ends. The object of using a thoroughly wet duster is twofold—firstly, it does its work more efficiently; and, secondly, it prevents any dust from flying about the room or falling on the floor or bed. Although under the careful conditions in which patients are kept in sanatoria the dust probably contains no tubercle bacilli as a rule, yet, supposing any should have been carried about the room by the air, it is well known that they can live for a very long time in dust, and when dry can be carried about by currents of air from place to place. When a wet duster is used, not only are the bacilli more readily taken up by the duster, but, being damp, they cannot be blown about. Secondly, dust, *per se*, is injurious to phthisical patients, and therefore the removal of all dust particles must be as thorough as possible.

### REST AND DINNER.

When the patients in bed have finished breakfast they will need either to be washed entirely or to be assisted in washing themselves. In all these particulars, of course, the doctor's orders will be followed. Some in-bed patients will be allowed to be up for a few hours daily, lying on the long couch which is an integral part of every patient's room. They will require to be settled comfortably, with books, papers, and any other requirements by the side of their couch or bed. Every room must be put clean and tidy by twelve o'clock, as at that time each patient goes to his room to rest for an hour before dinner. During this hour the patients are visited by the doctor, and the nurses are occupied with dressing and tidying themselves, and preparing the dinner trays for the patients who are in bed. Where the number of patients who are in bed are large enough it is a good plan for the head nurse (or other responsible person) to carve and send up the meals half-an-hour before the regular meal in the dining-room; but otherwise the food is served out by the doctor from the dining-room, and taken up by the nurses, who then return to their own dinners in the dining-room, at the same time as the patients. This plan, though unusual, has been found to work well in some sanatoria. After dinner, which takes from one-and-a-half to two hours, the trays are fetched away, and as many nurses as possible go off duty, and those who are on have usually a slack time till the time for preparing the in-bed patients for their supper and getting ready their trays. From six p.m. to bed time every nurse is generally fully occupied. Many patients have to be sponged all over before settling to rest, some require help with their baths, and all in-bed patients need assistance with washing and preparing for the night.

### FEEDING THE PATIENTS.

An important part of a nurse's duty is feeding the patients. In many severe cases of phthisis it is important to reduce the possibilities of fatigue as much as possible, and they must therefore be spared all unnecessary exertion. By feeding is meant actually putting the food into the patient's mouth. To some patients this is a vexation and annoyance, and requires to be done with the utmost care and tact; to many it is the greatest relief and boon. Many patients are helped by the presence of a nurse while they are eating; or, again, if the nurse reads aloud an amusing book it will often make the patient take his food with less pain and toil.

All these duties performed, the nurses are finally off duty between half-past nine and ten o'clock. The whole place is quiet, and everyone has retired to rest by ten.

### NO NIGHT DUTY.

In a sanatorium there is usually no need of any night nurse. Nurses must have their bedrooms in such a position that they are in easy communication with the patients under their charge, and all patients' bells must ring into the nurses' bedrooms. It is a wise plan to have one or two bedrooms quite away from the patients to which nurses may retire in turn, so as to have a spell of absolute off duty time for a night or two. With regard to times off duty. Everyone must be off duty for a short time every day, of course, but more important it is to give every nurse a chance of having 24 hours off duty once a month. The situation of a sanatorium is isolated, and the work apt to be monotonous, so that a sufficient time to enable a nurse to get into entirely different surroundings is advisable.

### Echoes from the Outside World.

AN OPEN LETTER TO A HOSPITAL NURSE.

VERY wisely, the Dean and Chapter of St. Paul's Cathedral abandoned the memorial service which had been arranged for Monday. So long as there is even the shadow of a shade of doubt respecting the Legations at Peking, it is permissible to cherish the hope that the Chinese authorities are not persistently lying; and, at any rate, Sir Halliday Macartney, the respected councillor and English secretary to the Chinese Legation in this country, would not assert that the foreign Ministers and families are safe unless he really believed it. His statement that the Ministers will very soon be on their way to join the allied forces at Tientsin, though founded entirely upon information derived from Chinese sources, is positive and startling enough to make one feel that a memorial service might have been premature. But the vast majority of people will not accept assurances that the massacre has not taken place unless, or until, they are countersigned by some of the supposed victims of Chinese cruelty and perfidy. If Sir Claude Mac Donald, or Sir Robert Hart, cannot for the moment be produced in person, an authentic communication from one or both of them must be obtainable, assuming that they are alive. Their silence is all the more inexplicable if they are safe, for a letter written by Sir Claude on the 4th does not count; and any number of Chinese edicts and explanations will not really modify the sad conviction which has been forced upon us, so long as they are entirely unsupported by European evidence. Meanwhile, it seems to me intensely significant that the Consuls at Shanghai have refused to meet Li Hung Chang at lunch, one of them candidly saying that he would never accept such an invitation until he knew the fate of the foreign Ministers at Peking. In London the probability of the members of the Chinese Embassy being requested to leave is discussed, but this will only happen in the event of actual war, which, at present, is not declared.

---

I AM not going to remark that the heat during the last week has been excessive, because the statement would scarcely be original, and most of you must have felt the truth of the assertion for yourselves; but there have been two or three interesting little points in connection therewith which are worth noticing. To begin with the highest in the land, did you see that our dear sensible Queen, notwithstanding her 80 years, wore a thin white dress, her figure half concealed by a white shawl, and a white hat with a white feather? Such was the attire in which she showed herself to the Christian Endeavourers at Windsor Castle upon the occasion of their visit. Hundreds of them went to see the home of England's Queen, buoyed up also with the hope that possibly they might get a peep of the great lady herself. After hours of waiting in the broiling sun came the reward. The Sovereign, having heard that the Christian Endeavourers were patiently standing on the chance of seeing her during her evening drive, sent out to say that she would be glad for them to come into the quadrangle opposite her oak dining room, and there they watched her, clad in cool white, get into her carriage with the help of her Indian attendant. She stayed whilst they sang "God Save the Queen," and a hymn to follow, and it is said that Her Majesty looked almost as pleased as her visitors.

---

To return to the hot weather. The Prince of Wales before the races on Friday stated that owing to the extreme heat he wished the frock coat and silk hat to be abandoned. Consequently, the men at Sandown were clothed much more rationally than usual, the Prince himself wearing a serge lounge coat, light trousers, a white bowler hat, and his favourite brown boots. In many of the suburbs the coachmen have been allowed to wear straw hats, but only a few have been seen in London upon the drivers, though I rejoice to note the increasing number of horses which are bonnetted. These bonnets are of all shapes and sizes. The most popular, because the most easily obtainable, is a sort of coarse rush hat, which can be bought in most shops for about threepence, and which fits on very nicely to the horse's head when the holes for the ears are carefully cut out. I was greatly amused at a scene I witnessed in a quiet street, and I wished I had been an artist and sketched the group as they stood. The owner of the establishment was fitting on "the first hat," so the picture might have been called. The mother, a grown-up daughter, and two little children around were making suggestions to the "milliner" and encouraging noises to the horse, who stood patiently still, only now and then shaking his head resignedly as if to say, "I suppose it is all right, but whatever are you at?" The smile of supreme triumph which broke over the faces of the family when the headgear was firmly fixed, was good to see. One of the omnibus companies has ordered a market gardener to send up a cartload of cabbage leaves every day as long as the hot weather lasts to keep their horses' heads cool; and considering the number who died or were incapacitated in one day alone last week, the preventive measures do not seem to have come too soon.

---

YOU all know the incident of Lord Roberts being discovered with a little Boer girl on his knee teaching her to read, and bidding the soldier who brought him a message go away, saying, "Can't you see I'm busy?" From a private letter I have just received, it is evidently not only the officers who are doing their best to show the Boers that, though our soldiers may be fierce enough upon the field, they are as tender as women when a little child is in question. My correspondent, who lives with her brother, a doctor, in a village in the Orange River Colony, lately visited by General Rundle's troops, writes: "This afternoon about half-past two a military waggon came down and stopped at our gate. It was accompanied by an escort of seven or eight soldiers, one ambulance man, and a kind of sergeant in charge. In the waggon was a Dutch woman and her six children, the eldest about seventeen, the youngest exactly five days old! One of the children, a little girl about eight, had a broken leg, and it was in order that the damage might be repaired that the family had been brought to the village. We went out to them, and found them excessively poor; the mother had nothing on but a chemise, a white petticoat, and a blanket, her other clothes having, she said, been sent to the wash. The soldiers told us that when she was taken the missing clothes were found in her possession in a little wet bundle. Of course we gave her some garments. It appears that the family had started to fly from the troops a week ago, and that as they had no vehicle of any kind they put their belongings on a horse, and, notwithstanding the condition of the mother, the family walked. In coming down a mountain the things on the horse's back slipped, and the animal started kicking. Somehow in the dark the little girl got under its feet, and her leg was broken. At last the troops found the family, took the father prisoner, and brought the rest here. It was so pathetic to see the poor little girl with the broken leg—unset for six days—carried in and out of the surgery by the soldiers. She clasped one small arm round the neck of each soldier, and put her pale, drawn face up against the rough cheeks of her nurses. The only attempt to give the leg 'first aid' had been to wrap it in a piece of sheep's skin. When she came out of the surgery she had only half-recovered from the effects of the chloroform, and lay quite limp in the big soldier's arms with both her own round his neck. When he got into the waggon he would not put her down or let anyone else have her, but nursed her all the way up to the house where the family were to stay for the night."

### Everybody's Opinion.

[Correspondence on all subjects is invited, but we cannot in any way be responsible for the opinions expressed by our correspondents. No communication can be entertained if the name and address of the correspondent is not given, as a guarantee of good faith but not necessarily for publication, or unless one side of the paper only is written on.]

### THE GENERAL HOSPITAL, MADRAS.

"A RESIDENT IN MADRAS" writes: In looking through the files of THE HOSPITAL "Nursing Mirror" I see an article in your issue of January 27th last on the subject of the difficulty in obtaining nurses for the General Hospital at Madras, and an intimation that the pay of the nurses has been increased. I think I am correct in saying that there is no difficulty in obtaining nurses, but a difficulty in keeping them, and I doubt whether the question of pay has anything to do with the difficulty. It is unnecessary, and probably undesirable, that I should enter into details on the subject of the management of the nursing staff of the Madras General Hospital, but I am satisfied that the question is one which deserves the attention of the Local Government.

### BABY AND INCUBATOR.

"FIRST EXPERIENCE" writes: As I know your paper is much read by maternity nurses I think a few particulars of the last little one of whom I had charge may be of interest. It was a little girl, a six and a-half months' child. She was put into one of Hearson's incubators on the second day after her arrival. On the fourth she weighed 3 lb. 7 oz. During the first, second, and third weeks the temperature of the incubator was kept at 85 degrees, and 9 oz. was gained in weight. During the fourth and fifth week the temperature was maintained at 80 degrees, and 6 oz. were gained. During the sixth and seventh weeks the temperature was lowered first to 78, then to 75 degrees, and 7 oz. and 5 oz. in weight were gained respectively. As the eighth week was to be the last of the baby's residence in the incubator the temperature was reduced to 70 degrees, and during the last two days the lid of the incubator was also kept open. On the last day of the eighth week the child was put into an ordinary lined cot, protected by curtains. She was fed with natural food at intervals of one and a half hours during the first three weeks, at first by means of a small teaspoon, later by tube and teat. During the fourth and fifth weeks food was given every two hours, and after then the natural food was supplemented by artificial. Three drops of brandy were given in the food every four hours. Every morning the baby was rubbed with salad oil and brandy in equal parts, and wrapped in cotton wool. She was not bathed or dressed till nearly eight weeks old. The first week after being out of the incubator she gained 4 oz., and is now, at three months old, a bright, pretty little child weighing 6 lb. 14 oz.

### OUR DUTY TO THE DYING.

"A LOVER OF NURSING" writes: How nice it was to see in your columns a patient's idea of our duty to the dying. It expresses my own very nearly. I am at present on night duty, nursing a sweet soul who will never be better on earth. She has, perhaps, been over-anxious to get well, not for her own sake, but for the sake of others who are largely dependent on her for much of the happiness of their lives. Sometimes during her long illness she has murmured, thinking that perhaps other and more successful means might have been used by the several doctors. When, however, a day or two since, after another consultation, the doctors found that nothing more could be done, we deemed it best to tell the patient. It was pathetic sadness that came into her face for a little while on hearing the hopelessness of her case; but since then that sadness has been replaced by resignation—not only so, but she is looking forward to the time when she shall exchange for her weariness and pain joy and gladness, for will she not be in the presence of the Master whom she has well loved? One may not always be able to tell a patient of the approaching end, but in every possible case how right it surely is. To my mind it need not be a dreadful message to deliver, looking at it in the truth of "Death being just the gate to Life." It need not be talked about in a mournful manner, for verily "It is not the whole of life to live," and if death means, as it should do, entering into a fuller, higher stage of life, then why not let our patients get all the comfort they can from such thoughts? for much comfort and peace surely may be had from realising that death is only an episode, happening in the life of each one of us, which launches us into the greater life eternal. Then, again, who of us has not come across cases of sick folk who, knowing their days were numbered, have hastened to forgive old grievances or set to rights little misunderstandings, which just permit the stories of some lives to have happy rather than other endings?

### Appointments.

WEST HAM HOSPITAL, STRATFORD.—Miss A. E. Ough has been appointed Matron. She was trained at West Ham Hospital, and has since been staff nurse, out-patient nurse, sister in charge of two male wards, operating theatre, and casualty room in the same institution.

### Minor Appointments.

NEW INFIRMARY, SELLY OAK.—Miss Alice Edgcumbe Chevallier and Miss Eleanor Taylor have been appointed Charge Nurses. Miss Chevallier was trained at Crumpsall Infirmary, Manchester, and has since been charge nurse at Prescot Union Infirmary. Miss Taylor was trained at Chorlton Infirmary, and has since been charge nurse at the Union Infirmary, Salford, and other infirmaries.

HUNSLET UNION WORKHOUSE INFIRMARY.—Miss Elizabeth Gott Scaife has been appointed Charge Nurse. She was trained at York Home for Nurses, and has since been district and private nurse, charge nurse at South Shields Union, charge nurse at Wharfedale Union, and district nurse in connection with Apperley Bridge District Nursing Association.

ENFIELD ISOLATION HOSPITAL, WINCHMORE HILL.—Miss Katharine Farringhton has been appointed Night Superintendent. She was trained at the Royal Alexandra Hospital, Brighton, and the Victoria Hospital, Folkestone, and has since been temporary sister at Bagthorpe, Nottingham, and staff nurse at Allt-yr-yn Hospital, Newport, Mon.

DUMFRIES AND GALLIVRAY ROYAL INFIRMARY.—Miss Mary L. Potter has been appointed Night Superintendent Nurse. She was trained at Paddington Infirmary for three years, and has since been staff nurse at the Royal Infirmary, Edinburgh, and sister at Poplar and Stepney Asylum. From 1897 she has been training at Guy's Hospital.

NURSES' HOME, WHISTON WORKHOUSE INFIRMARY.—Miss Annie Wood has been appointed Home Sister. She was trained at the Royal Infirmary, Manchester, and has since been staff nurse in the same institution, and charge nurse at Prescot Union Infirmary.

COLCHESTER HOSPITAL.—Miss Ethel M. Barchard has been appointed Sister. She was trained at University College Hospital, London, and holds the L.O.S. certificate.

### Wants and Workers.

WILL a nurse kindly give her old uniform to a married nurse who is compelled to commence work again, and finds it difficult to buy suitable cloth? About 100 recent copies of THE HOSPITAL offered in exchange.

MISS M. E. MAINGAY, Havelet, Florence Road, Boscombe, sends many thanks to those friends who have so kindly helped the invalid nurse. She will be very grateful if a few more will offer to make up the rest of the sum still required, by guaranteeing 1d. per week each.

### For Reading to the Sick.

"DOUBT not, therefore, but earnestly believe."

There is no unbelief !
Whoever plants a seed beneath a sod
And waits to see it push away the clod,—
He trusts in God.
Whoever says, when clouds are in the sky—
"Be patient, heart; light breaketh by and by,"
Trusts the Most High.
Whoever sees, 'neath winter's field of snow,
The silent harvest of the future grow,
God's power must know.
Whoever lies down on his couch to sleep,
Content to lock each sense in slumber deep,
Knows God will keep.
There is no unbelief !
And day by day and night unconsciously,
The heart lives by that faith the lips deny—
God knoweth why.

**Reading.**

"Everything which is, is a revelation of God." All nature is the disclosure of God. "The heavens declare the glory of God, and the firmament sheweth His handywork." "The invisible things of Him since the creation of the world are clearly seen, being perceived through the things that are made, even His everlasting power and divinity." "There is no creature," says the author of "The Imitation of Christ," "so small and abject, that it representeth not the goodness of God." The natural world testifies to God—to His existence as its Maker, to His wisdom, power, order, and beauty.

"How do you know," a Bedouin was asked, "that there is a God?" "In the same way," he replied, "that I know, on looking at the sand when a man or beast has crossed the desert—by His footprints in the world around me."—*Liddon.*

"One who believes in the God of Christianity is bound to believe that creation is His work from end to end, that it is a rational work, and the work of a Being who is wholly good. He is bound to believe that 'God's mercy is over all His works,' that 'not a sparrow falls to the ground, without His knowledge, that there is design and purpose everywhere. But he is not bound to know, or to say that he knows, what that purpose is, or to show that marks of beneficence are everywhere apparent."—*Aubrey Moore.*

Once more, O Lord, behold me at Thy feet.

Thou art the very life which beats within my heart:
I have no power to choose: from Thee I cannot part.
O Light of all the world, that gladdened weary eyes !
Didst Thou to darkness sink, never again to rise?
O Voice, more sweet than men had known on earth before!
Has Thy strange music died to silence evermore?
O Death, thro' which we dreamed of gain in utter loss!
Was it indeed defeat, that passion of the Cross?
Then—Brother, Master, King!—I take my part with Thee,
And where Thou art, O Lord, there let thy servant be.
The awful unknown Power that in the darkness lies,
Thou saidst could be revealed, thro' Thee, to mortal eyes:
And what tho' earth and sea His glory do proclaim,
Tho' on the stars is writ that great and dreadful name—
Yea—hear me, Son of Man—with tears my eyes are dim,
I cannot read the word which draws me close to Him.
I say it after Thee, with faltering voice and weak,
"Father of Jesus Christ,"—this is the God I seek.
And can it be that *Thou* mistook'st that name divine?
Then let me share Thy dream, my error be like Thine.
On Thee I lean my soul, bewildered, tempest-tost,
If Thou can'st fail, for me then everything is lost.
For triumph, for defeat, I lean my soul on Thee;
Yes! where Thou art, O Lord, there let Thy servant be.

—*Anon.*

### Notes and Queries.

THE Editor is always willing to answer in this column, without any fee, all reasonable questions, as soon as possible.

But the following rules must be carefully observed:—

1. Every communication must be accompanied by the name and address of the writer.
2. The question must always bear upon nursing, directly or indirectly.

If an answer is required by letter a fee of half-a-crown must be enclosed with the note containing the inquiry.

*Obstetric Nursing.*

(163) May I ask if you will kindly inform me whether Western Australia would be a good field for obstetric nurses? 2. Also when will it be likely that the Midwives' Bill will get its third reading in Parliament? 3. May I also ask if there is any magazine published specially for midwives?—*M. B.*

1. Not unless you have friends there to introduce you. 2. This Bill has already found a place among the slaughtered innocents. 3. "Nursing Notes" devotes considerable space to this section of nursing. It is to be obtained monthly from 12, Buckingham Street, Strand, price 2d.

*Patent.*

(164) Will you please tell me how to get an invention patented, and the probable cost; and (2) if any firm would buy it and patent it for me if the cost is too much?—*Comfort.*

To patent an invention is a tedious and costly process, and moreover it is one of doubtful success, unless the patentee has money to advertise and push the sale of the article after the letters are granted, but a provisional protection does not cost much, and the forms can, we believe, be procured at the post office. 2. You can only find out this by placing the matter before likely firms. Do not trust to agents.

*Pelvis and Skull.*

(165) Will you kindly tell me where I can hire a female pelvis and fœtal skull and what would be the probable cost?—*E. A. D.*

The secretary of the Midwives' Institute, 12, Buckingham Street, Strand, might possibly know of someone who could lend you the skull and pelvis privately, or you might apply to your local anatomist to procure them for you. See "Sale and Exchange" column in "Mirror." A set was advertised on June 9th.

*Employment.*

(163) Can you kindly tell me how a monthly nurse (certificated) just from the hospital can get cases if she does not know of any doctor to ask? Is it any use to advertise, and in what paper? I should be so glad of a hint.—*Nurse Edith.*

Maternity nurses frequently advertise in the medical and ladies' journals. See similar advertisements in "Mirror." There can be no harm in calling or leaving a professional card on the medical practitioners in the neighbourhood.

*Probationers.*

(167) Can you kindly tell me of a private institute or nursing home in Birmingham where they take probationers and send them to a general hospital for training, giving them a small salary and afterwards taking them on to their private staff?—*S. T.*

The Birmingham and Midland Counties Training Institution for Nurses, 12, The Crescent, Birmingham, appears to answer your requirements. Apply the Lady Superintendent.

*Rome or Nice.*

(168) Will you kindly give me the address of a private nursing institution in Rome or Nice? I wish to make inquiries as to terms of engagement with a view to joining.—*G. G.*

The Hollond Institute of English Trained Nurses has a branch in Nice. Address 1, Tavistock Chambers, Bloomsbury, W.C. There are two or three homes for English nurses in Rome. Apply English Nursing Sisters, Piccola Campagnia di Maria, 45, Via Castelfidardo, or Miss Martin, St. Paul's Home, 62, Palestro.

*Lupus.*

(169) I shall be much obliged if you will kindly inform me (1) whether there is such a thing as electric light treatment for lupus, and if so, where I could obtain information with regard to it; (2) I should also be much obliged if you could give me the name of a book recently published by the Liverpool School for Tropical Diseases. I think the title was "On the Prevention of Malarial Diseases," or something to that effect.—*Sussex.*

(170) I am suffering from tubercular lupus, and have had my face and neck scraped at intervals at Addenbrooke's Hospital, Cambridge, and now I am told there is a certain institute in Denmark where they effect a cure by a certain light. Could you tell me if there is any hospital in London where they use this light for the cure of this disease, as I could not afford the journey to Denmark. I may state I am a nurse, but in consequence of having this disease, have been unable to follow my profession for the past twelve months.—*M. C. F.*

Arrangements have quite recently been made at the London Hospital for carrying out the treatment of lupus by concentrated light in accordance with the method of Dr. Finsen. The book you refer to is doubtless "Instructions for the Prevention of Malarial Fever for the Use of Residents in Malarious Places." It is published by the University Press, Liverpool.

**Standard Books of Reference.**

"The Nursing Profession: How and Where to Train." 2s. net.; post free 2s. 4d.
"The Nurses' Dictionary of Medical Terms." 2s.
"Burdett's Series of Nursing Text-Books." 1s. each.
"A Handbook for Nurses." (Illustrated.) 5s.
"Nursing: Its Theory and Practice." New Edition. 3s. 6d.
"Helps in Sickness and to Health." Fifteenth Thousand. 5s.

All these are published by THE SCIENTIFIC PRESS, Ltd., and may be obtained through any bookseller or direct from the publishers, 28 & 29, Southampton Street, London, W.C.

